# Detecting Dementia from Face-Related Features with Automated Computational Methods

**DOI:** 10.3390/bioengineering10070862

**Published:** 2023-07-20

**Authors:** Chuheng Zheng, Mondher Bouazizi, Tomoaki Ohtsuki, Momoko Kitazawa, Toshiro Horigome, Taishiro Kishimoto

**Affiliations:** 1 Graduate School of Science and Technology, Keio University, Yokohama 223-0061, Kanagawa, Japan; 2 Faculty of Science and Technology, Keio University, Yokohama 223-0061, Kanagawa, Japan; mondher.bouazizi@gmail.com (M.B.); ohtsuki@keio.jp (T.O.); 3 School of Medicine, Keio University, 35 Shinanomachi, Shinjuku-ku, Tokyo 160-8582, Japan; m.kitazawa@keio.jp (M.K.); toshirou.ho@keio.jp (T.H.); tkishimoto@keio.jp (T.K.)

**Keywords:** dementia detection, face mesh, action unit, HOG, machine learning

## Abstract

Alzheimer’s disease (AD) is a type of dementia that is more likely to occur as people age. It currently has no known cure. As the world’s population is aging quickly, early screening for AD has become increasingly important. Traditional screening methods such as brain scans or psychiatric tests are stressful and costly. The patients are likely to feel reluctant to such screenings and fail to receive timely intervention. While researchers have been exploring the use of language in dementia detection, less attention has been given to face-related features. The paper focuses on investigating how face-related features can aid in detecting dementia by exploring the PROMPT dataset that contains video data collected from patients with dementia during interviews. In this work, we extracted three types of features from the videos, including face mesh, Histogram of Oriented Gradients (HOG) features, and Action Units (AU). We trained traditional machine learning models and deep learning models on the extracted features and investigated their effectiveness in dementia detection. Our experiments show that the use of HOG features achieved the highest accuracy of 79% in dementia detection, followed by AU features with 71% accuracy, and face mesh features with 66% accuracy. Our results show that face-related features have the potential to be a crucial indicator in automated computational dementia detection.

## 1. Introduction

Dementia is a debilitating and progressive neurodegenerative disorder that affects an individual’s cognitive abilities and daily functioning. It is a common condition among older adults, with an estimated 55 million people living with dementia worldwide and 10 million new cases every year [1]. Dementia is characterized by a decline in cognitive function, such as memory loss, language impairment, disorientation, and difficulties with executive functions (such as planning, organizing, and decision-making). As the disease progresses, individuals with dementia may also experience behavioral and psychological symptoms such as depression, anxiety, agitation, and aggression [2]. As a contested term, dementia relates to a group of diseases instead of a specific type of disease [3]. By now, it has more than 200 subtypes that do not share the same path or process [4]. The most common cause of dementia is Alzheimer’s disease (AD), which accounts for approximately 60–70% of cases [1]. Other types of dementia include vascular dementia, Lewy body dementia, frontotemporal dementia, mixed dementia, etc.

Typically, AD progresses along three stages: early, middle, and late, which sometimes are also referred to as mild, moderate, and severe. In the early stage of AD, people can live independently but may suffer symptoms such as subjective memory loss, and forgetting familiar words or locations. The middle stage of AD usually lasts for years and maybe the longest stage during the progression, during which the patient will require heavier care from others. They may experience forgetting events or personal history, being confused about what day it is or where they are, etc. The symptoms become severe in the late stage, and patients gradually become unable to respond to the environment, have a conversation, or even control their movements [5].

Age is widely recognized as the most significant risk factor for dementia. The world population is growing older at an unprecedented rate. According to the United Nations, the global population aged 65 years or over is projected to reach 1.5 billion by 2050, up from 703 million in 2019. This represents a significant demographic shift, with older adults expected to account for more than 20% of the world’s population by mid-century [6]. In some countries, such as Japan and Italy, the aging of the population is even more pronounced, with over a quarter of the population aged 65 or over.

According to the Alzheimer’s Association, 1 in 9 people aged 65 or older has dementia. As of 2023, 6.7 million Americans are suffering from dementia, with 73% of them aged 75 or older. As the elderly population in the United States continues to increase, there will be a corresponding rise in both the number and percentage of individuals who suffer from Alzheimer’s or other types of dementia. Without the discovery of medical advancements to prevent or treat AD, it is estimated that the number of people aged 65 and older with AD could potentially reach 12.7 million by the year 2050 [7]. The World Health Organization (WHO) reported, in a study [8] where the status of subjects was tracked over years, that the annual incidence of dementia is between 10 and 15 cases per thousand people. Patients who developed dementia have an average of 7 years of life expectancy, and no more than 3% of them will live longer than 14 years. This problem is compounded by the fact that current medicine has no effective treatment to cure AD [9]. That being said, certain lifestyle changes and treatments can slow down the progression of AD and improve the quality of life for those living with AD [10]. Early diagnosis of dementia allows patients to receive appropriate treatments and suggestions to slow down or even halt the progression of the disease. By developing an efficient early detection system for dementia, many patients’ life quality can be significantly improved and even many lives can be saved. These facts urge institutions and researchers to pay attention to the development of methods for the prevention and early detection of dementia.

Traditional methods for diagnosing dementia, such as brain scans and psychiatric tests, face significant limitations that prevent their mass deployment. Firstly, these methods are resource-intensive and costly, requiring specialized equipment, trained personnel, and extensive time commitments. As a result, it becomes challenging to scale up these diagnostic procedures to reach a larger population in need. Additionally, the invasive nature of brain scans and the comprehensive nature of psychiatric evaluations can deter individuals from willingly participating in these tests, leading to low uptake and potential delays in diagnosis. Furthermore, the expertise required to interpret and analyze the results of these tests is often concentrated in specialized healthcare settings, limiting access to diagnosis for individuals in remote or underserved areas. Given these factors, the widespread adoption of traditional diagnostic methods for dementia becomes impractical and underscores the need for more accessible and efficient alternatives.

A cost-effective and scalable screening method is needed to detect subtle indicators of dementia, such as subjective memory loss, Mild Cognitive Impairment (MCI), and AD. MCI is a condition characterized by cognitive changes that are noticeable and measurable but not severe enough to meet the criteria for a diagnosis of dementia. It is often considered an intermediate stage between normal aging and dementia. Language is an important indicator in the detection of dementia because AD significantly impairs patients’ language abilities. These impairments are easily revealed in certain tasks, such as describing pictures. In picture description tasks, dementia patients and healthy individuals describe the same image differently. Dementia patients show more grammar mistakes, use shorter sentences, and struggle with word finding and sentence organization. They may have difficulty understanding the connections between events in the picture, indicating the involvement of both visual and language abilities in accurate descriptions. As a result, many speech-based dementia detection methods have been proposed [11].

Compared to the extensive research on speech and language-based dementia detection, facial features have received much less attention, even though facial features have proven to be effective indicators in dementia detection [12]. Previous works [12,13] have made some explorations on dementia detection using facial features or facial images. However, these works only perform experiments with raw facial images or some facial features. The dataset used in their experiments is relatively small and limited, which could also introduce bias.

## 2. Related Work

In dementia, brain regions such as the hippocampus (involved in memory), language centers, frontal lobes, and communication pathways can be damaged, leading to language and memory problems. Many researchers have paid attention to speech data, and many methods were developed to distinguish dementia patients from healthy subjects [14,15,16,17,18,19]. These methods mainly used natural language processing techniques to evaluate the data. Different features were extracted and different types of machine learning models were proposed to perform classification on the text data, with labels indicating whether the text samples came from a dementia patient or a healthy subject. Weiner et al. [14] compared two feature extraction pipelines for dementia detection. The first pipeline involves manual transcriptions, while the second pipeline uses transcriptions created by automatic speech recognition (ASR). According to the study, the transcription quality of the ASR system is a dependable feature for detecting dementia on its own. Moreover, the features extracted from the automatic transcriptions exhibit comparable or slightly superior performance when compared to those derived from manual transcriptions. Mirheidari et al. [15] investigated how word vector representation models perform in detecting signs of dementia. Motivated by the fact that dementia patients suffer from impairment in accurately expressing something, they analyzed conversations designed to test examinees’ long-term and short-term memory. Three methods were proposed to show the potential of word vectors in a classification task. Their study concluded that it is possible to detect signs of dementia with a speech recognizer, even though the recognition result has a high error rate. Zhu et al. [16] found that transfer learning models utilizing text data outperform those using audio data, likely due to the high similarity between the pre-training text dataset and the Cookie-theft picture text dataset. Multi-modal transfer learning shows a slight improvement in accuracy, indicating that audio and text data provide limited complementary information. However, multi-task transfer learning results in limited improvements in classification and a negative impact on regression. They also identified that inconsistencies between AD/non-AD labels and Mini-Mental State Examination (MMSE) scores can limit the performance of multi-task learning. Mahajan et al. [17] re-implemented NLP methods that utilized Convolutional Neural Networks (CNN) and Long Short-Term Memory (LSTM) architectures and specific conversational features to diagnose AD. They examined why these models had lower accuracy on the ADReSS [20] dataset than the DementiaBank [21] dataset. Additionally, they created a deep learning-based solution using Recurrent Neural Networks (RNNs) that performed binary classification of AD from natural speech. Their method combined acoustic features using Speech-GRU, improving accuracy by 2% over the acoustic baseline. Furthermore, when enriched with some targeted features, their method outperformed acoustic baselines by 6.25%. They proposed a bi-modal approach to AD classification and discussed the potential benefits of their method. Shibata et al. [18] adapted and applied the idea density to the Japanese language. They defined several rules to count the idea density in Japanese speech and proposed a method to evaluate the idea density using machine translation. They proposed different pipelines for estimating the idea density, and compared the performance of different idea density calculation methods. They concluded that these findings prove that it is feasible to apply the method in the area of dementia detection. Santander-Cruz et al. [19] proposed a method in which they extracted 17 kinds of features, which represented syntactic, semantic, lexical, and demographic information of the speech samples collected in the DementiaBank Pitt Corpus dataset. The relevance of the 17 features was quantified by calculating a mutual information score that can represent the dependency between the extracted features and the MMSE score. They concluded that their methodology is superior to the methods based on syntactic information or the BERT method with only linguistic features. Farzana et al. [22] evaluated how removing the disfluency in the annotated result of an automatic speech recognition system affects the performance of dementia detection. They used an existing tool that detects and tags disfluency in transcriptions of speech. Through a set of experiments, they found that removing the disfluency has a bad influence on dementia detection performance, which reduces the detection accuracy. Ilias et al. [23] proposed a novel method that combined two modalities such as speech and transcripts into one model based on the vision transformer. They used the gated multimodal unit to control how much influence each modality had on the final classification. They also used crossmodal attention that learned the relationships between modalities in an effective way. They tested their work on the ADReSS Challenge dataset and proved their model’s superiority over existing methods.

The above-mentioned most recent studies concentrate on the use of speech and text data to detect dementia. However, in some past studies [24,25] which analyzed the facial expressions of dementia patients, some conclusions mentioned that some deviations happen among dementia patients. The different displays of facial expressions have the potential to detect dementia. Asplund et al. [24] showed that patients with some types of dementia tend to show fewer facial expressions. They analyzed the ability to produce expressions using the Facial Action Coding System (FACS) under pleasant and unpleasant stimulus conditions, while, in their later study [25], they compared two methods developed for interpreting the facial expressions of demented people. In this study, disagreement between the two methods was reported, and demented people had less clarity or a lower amount of facial cues. Based on the findings of this research, we can notice that the facial expressions of demented people deviate, which may function as indicators in dementia detection.

In recent years, some works [12,13,26] collected facial expression data from dementia patients and explored the potential of using facial expressions to detect dementia. Tanaka et al. [12] collected human-agent interaction data of spoken dialogues from 24 participants. They extracted facial features from these data and used L1-regularized logistic regression to classify dementia patients and healthy subjects. Their research identified several features, including Action Units (AUs), eye gaze, and lip activity, as contributing to the classification. They also identified the importance of each feature in their L1 regularized logistic regression. The majority of the important features are AU-derived features. However, their methods do not apply to natural free conversation, nor did they evaluate their methods on a large dataset. Umeda et al. [13] examined whether artificial intelligence could distinguish between the faces of people with and without cognitive impairment. They achieved a classification accuracy of 92.56%. Their study showed that it is possible for deep learning to distinguish the faces of dementia patients from those of people without dementia. Although their study achieved high accuracy, they used facial images for the study, and institutional bias was reported [13]. Institutional bias can mislead the machine learning model so that the model learns environmental information instead of the dementia indicators.

Some papers have analyzed the expressions of dementia patients under controlled conditions. However, they did not apply them to the detection of dementia: Liu et al. [26] investigated how different sound interventions affect the facial expressions of older people with dementia. They showed the participants different sound pieces and analyzed their emotions. Jiang et al. [27] used a computer vision-based deep learning model to analyze facial emotions expressed during a passive viewing memory test in people with cognitive impairment caused by AD and other causes. Their results showed evidence that facial emotions are different in patients with cognitive impairment. Specifically, patients with cognitive impairment showed more negative emotional expressions and more facial expressiveness than healthy controls. They concluded that facial emotions could be a practical tool for screening patients with cognitive impairment.

As the above introduced works show, much research on dementia detection has paid attention to the exploitation of speech and text data, especially the DementiaBank dataset, and its derivative, the ADReSS dataset [20], which have been extensively studied and investigated. However, some researchers found that patients who have dementia behave differently from healthy subjects in facial expressions [24,25]. At the same time, the video data form a rich and informative source from which we can easily extract facial features. Motivated by these facts, we decided to explore the potential of facial features in dementia detection.

The use of facial features to detect dementia offers several advantages over other screening methods. The traditional diagnosis of dementia involves a range of tests, including brain scans and psychiatric evaluations. Unfortunately, these procedures can be highly stressful and time-consuming for patients. This not only adds to the overall burden on individuals, but also contributes to potential delays in diagnosis and subsequent treatment. Moreover, many patients may harbor reluctance or hesitancy to undergo these tests. The apprehension associated with the invasive nature of brain scans and the scrutiny of psychiatric evaluations can discourage individuals from seeking timely diagnosis and appropriate medical intervention. In recent times, speech-based detection of dementia has gained significant attention as a viable alternative. However, this approach still relies heavily on the use of the Boston Cookie Theft Test, a well-established tool for assessing cognitive function in individuals suspected of having dementia. Gathering speech data necessitates patients actively and intentionally describing a depicted scene, typically with the assistance of trained instructors. Consequently, this process remains costly, time-consuming, and limits the feasibility of large-scale speech data collection for diagnostic purposes. It is crucial to acknowledge the challenges faced by patients during the diagnostic process for dementia. Anxiety and confusion often accompany the tests they undergo, exacerbating the difficulties experienced by individuals already grappling with the potential cognitive decline associated with the condition.

While traditional methods such as psychiatric tests or brain scans can be stressful for the patients, the process of collecting facial features can be smoother and easier for the patients. It does not require the patients to undergo difficult tests which may be stressful for dementia patients who might feel reluctant and incompetent to fulfill such tests. Furthermore, facial feature data can be collected concurrently with other tests or screenings, eliminating the need for additional appointments and reducing the burden on patients. Facial analysis can not only function as a stand-alone biomarker for dementia detection, but can also be used in conjunction with other types of data, such as speech or brain scans, to provide a more comprehensive assessment of cognitive function. This complementary approach may lead to more accurate and reliable diagnoses, ultimately improving patient outcomes. Therefore, in this work, we are motivated to investigate the potential of facial features in dementia detection.

In this paper, we propose to use facial features such as face mesh, Histogram of Oriented Gradients (HOG), and AUs to detect dementia. Compared with raw face images, these features can remove the bias introduced by different lighting conditions or different environments in data collection. They can also anonymize the data to protect patient privacy. We extracted these different sets of features from the PRoject for Objective Measures using computational Psychiatry Technology (PROMPT) dataset https://www.i2lab.info/resources (Accessed date: 12 July 2023) [28] and investigated their effectiveness using different models and methods, such as the LSTM network and the Support Vector Machine (SVM). The PROMPT dataset consists of more than 60 h of video data. With such a relatively large dataset, we conducted experiments to investigate the effectiveness of the facial features mentioned above.

In exploring the connection between face and cognition, research can be categorized into either type I—Man as the measurement object, or type II—Man as the measurement instrument. Many works [29,30,31,32,33,34] have investigated face perception and cognition. Their research falls into type II, where Man functions as a measurement instrument. In their studies, subjects were required to complete a series of questions or tasks. In these questions or tasks, Man’s faces were shown to the subjects, after which subjects made a response to those faces—Man’s faces were used as the instrument to measure subjects’ latent ability. However, our work did not use Man’s faces as the measurement instrument; instead, the faces themselves (and the corresponding subjects’ cognitive status) were the measurement objects.

The main contributions of this paper are as follows:

- We investigated and analyzed the potential of using face mesh, HOG features, and AUs in dementia detection. These features have many advantages, such as the ability to remove the bias in the dataset and anonymize the dataset to protect patients’ privacy;
- We proposed several methods and pipelines to normalize and process these features and to detect signs of dementia using the facial features;
- We adapted and improved the method in [12] through segmentation and voting. We validated this method on a relatively large dataset—i.e., the PROMPT dataset.

The structure of the paper is organized as follows this: Section 3 introduces the dataset and the features employed in the experiments; Section 4 explains the settings and parameters of the experiments; Section 5 shows the results of the experiments of different features under different methods; Section 6 gives the conclusion and future directions.

## 3. Dataset and Facial Features

### 3.1. The PROMPT Dataset

The PROMPT dataset was collected by Keio Medical School. The dataset contains data collected from healthy subjects, dementia patients, bipolar disorder patients, and depression patients. The participants in the PROMPT dataset were diagnosed according to the Diagnostic and Statistical Manual of Mental Disorders, Fifth Edition (DSM-5) criteria by professional doctors. The data from dementia patients were used in this study. The dementia patients at Keio Hospital underwent an interview with the doctor. During the interview, the patients were asked to perform several language tasks. Their speech and images during the interview were recorded with a camera and two microphones separately.

- Free talk: In the free talk task, patients had casual conversations with the interviewer. Although it was called free talk, the interviewers asked the patients questions according to a prescribed list of questions, such as “Did you sleep well last night?” and “Was it hot or cold while you were sleeping?”;
- Q&A: In the Q&A task, the interviewer asked the patients three questions in sequence. The first question was “What would you do if there was a fire?”. The second question was “What would you do if you lost the umbrella you borrowed from others?”. The third question was “What would you do if you found an envelope with an address on it?”;
- Picture description: In the picture description task, the interviewer asked the patients to describe a picture shown to them.

The video data collected in the free talk task were used as the dataset in the experiments. Patients were recorded using a Real Sense camera placed on a table in front of the patients, with the upper body of the patients captured in the videos. The videos were stored as a sequence of images in “*.JPEG” format. A total of 447 videos provided by 117 subjects were recorded in the dataset. A single subject could have undergone the interview several times and provided more than one video recording. Each video recording lasted approximately 10 min. We used some tools to extract the face mesh data, HOG features, and AUs from the video data. These features are described and explained in the following sections.

### 3.2. Facial Feature: The Face Mesh

A face mesh is a digital representation of the geometry of a human face, typically created using 3D computer graphics techniques. It consists of a collection of interconnected vertices, or points in space, that are arranged in a mesh-like pattern to approximate the shape of the face. The face mesh is often used in computer vision and graphics applications, such as facial recognition, animation, and virtual reality, to track and manipulate the movements of the face in real time. By using a face mesh, it is possible to capture and analyze the subtle changes in facial expressions that convey emotion and communication cues, which can be useful in various fields such as psychology, marketing, and entertainment. With face mesh, it is possible to create a detailed 3D model of a patient’s face without capturing any personally identifiable information, such as facial features or skin color. This allows medical professionals to analyze and track facial movements and expressions without compromising patient privacy. Additionally, the use of face mesh can be particularly useful in telemedicine applications, where patients may not be able to visit a healthcare provider in person. By using face mesh technology, doctors and other healthcare professionals can remotely assess a patient’s condition and make informed diagnoses, all while ensuring patient privacy and security. Overall, the use of face mesh in medical applications offers a powerful combination of accuracy and privacy protection, making it an ideal tool for healthcare professionals who want to deliver the best possible care while preserving patient confidentiality.

**Figure 1:**
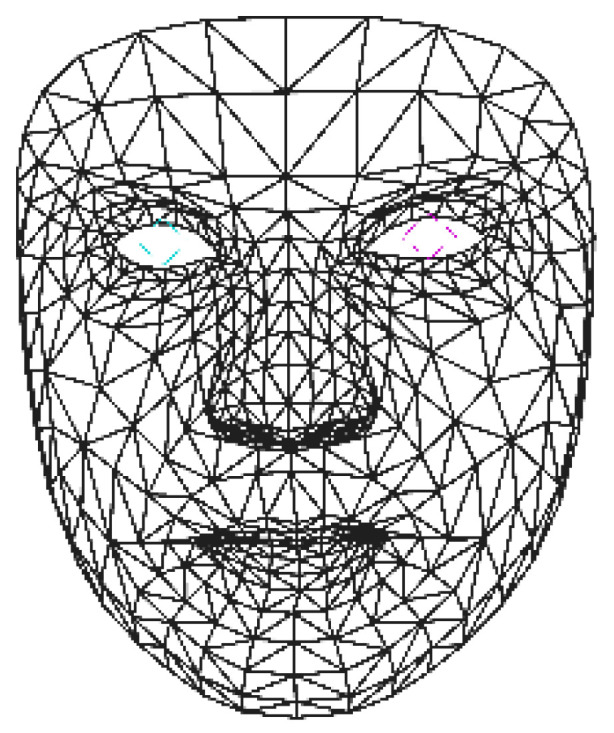
An example of face mesh extracted using MediaPipe. The blue and pink lines mark the positions of pupils.

MediaPipe [35] uses a combination of machine learning and computer vision techniques to extract a detailed face mesh from an input video stream or sequences of videos. The face mesh extracted by MediaPipe consists of a total of 478 vertices that are located at key points of a human face from each frame. These vertices are interconnected to approximate the 3D facial surface of the face in the given image. An example of a face mesh extracted by MediaPipe is shown in Figure 1.

### 3.3. Histogram of Oriented Gradients

HOG is a widely utilized feature descriptor in computer vision for detecting objects. It extracts information from an image by dividing it into small cells and computing a HOG within each cell. This process captures data about the local edge orientation and magnitude, which are then used to describe the appearance of an object. The HOG descriptor is able to capture these features without being affected by changes in illumination and provides a compact representation of the object that can be utilized for object classification or detection. The HOG feature descriptor has been applied with great success in various fields, including, but not limited to, pedestrian detection, face recognition, and object tracking.

HOG has been shown to be a useful tool in medical imaging analysis, specifically, in identifying and detecting various structures of interest, such as tumors or lesions [36,37]. By extracting information from images, HOG can provide a unique feature descriptor that can help distinguish between different types of structures. Additionally, HOG is invariant to changes in geometric and photometric transformations, making the HOG a robust feature.

### 3.4. Facial Feature: Action Unit

AUs are a standardized set of facial muscle movements used to describe and quantify different facial expressions [38]. Developed by psychologists Paul Ekman and Wallace Friesen in the 1970s, AUs offer a systematic and objective approach to measuring changes in facial expressions. There are currently 43 AUs that correspond to specific muscle movements, and they are widely applied in fields such as psychology, neuroscience, and computer vision to study emotional expression, communication, and social interaction. The use of AUs in analyzing facial expressions can provide valuable insights into how people perceive and respond to emotions [39]. Moreover, AUs have been incorporated into technology such as facial recognition software and virtual assistants, enabling these systems to interpret human emotions and provide a more natural and empathetic user experience.

Each AU is represented by a number, which corresponds to the intensity of the movement on a scale from 0 to 5, where 0 means the muscle is at rest and 5 means the muscle is fully contracted. The values of each AU can be combined to describe different facial expressions, such as a smile, a frown, or surprise. The AUs are formulated based on the anatomical location of the muscles involved in the movement. For example, AU 12 corresponds to the contraction of the zygomatic major muscle, which is located in the cheek and lifts the corners of the mouth, resulting in a smile. Another example is AU 2, which corresponds to the contraction of the frontalis muscle, located in the forehead, and is associated with raising the eyebrows in surprise or confusion.

## 4. Experiment Designs and Settings

In the following sections, we will explain the design and settings of our implementation. We used different processing techniques, models, and cross-validation schemes for different types of features to optimize the classification performance.

We used LSTM to process the data. At each step of the LSTM network, we input each frame of the data into the LSTM network. In the following parts, the experiment settings for each type of feature are described and explained, respectively.

### 4.1. Experimental Settings for Face Mesh

We utilized MediaPipe to analyze the face mesh data from the 447 videos in the PROMPT dataset’s free speech section. These videos typically have a duration of around 10 min and a frame rate of 30 frames per second. MediaPipe labels each frame in the video with information about the face mesh. Consequently, we converted each video into a collection of face mesh data consisting of 18,000 frames. Each frame of face mesh data included 478 landmarks, which were annotated with (x,y,z,v) values. Here, (x,y,z) represented the three-dimensional coordinates of each landmark, and *v* indicated whether the landmark was visible or not.

MediaPipe does not adjust or standardize the positioning or size variations of the face mesh data obtained due to differences in camera settings, such as angles or distances. For each input image, the face mesh was annotated at the exact location where the face was located in the image. Therefore, to reduce the influence of such factors on the classification, we normalized the face method in the following way.

**Figure 2:**
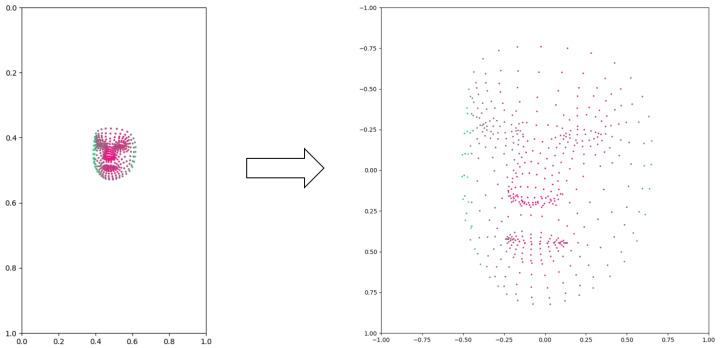
An example of face mesh before and after normalization. The color of the points indicates the depths (*z* coordinates), with red points closer to the camera and green points further to the camera.

First, coordinate transformation was applied to the coordinates of every landmark of a face mesh. In the extraction, the face in the original image and the annotated face mesh share the same coordinates. The faces can possibly appear anywhere in the image, and so does the face mesh. To make the LSTM network better learn the patterns of the face mesh, we remapped the coordinates of each landmark by transforming them with respect to a specified landmark in the center of the face. This makes this landmark the new origin of the coordinator and the rest of the coordinates are depicted with the new origin. After the coordinate transformation normalized the movement of the face mesh, we resized the coordinate range of the face mesh to [−1,1]. As the videos sometimes were recorded at different distances from the patient, the size of the face in the video may have varied due to perspective. Resizing the coordinate range ensured that the face mesh of each individual data was always kept at the same size. In our normalization process, we performed linear conversions on the coordinates of the face mesh while keeping the actual mesh unchanged. The purpose of normalization was to transform the coordinates and standardize their values, ensuring consistency across different samples. It is important to note that the normalization process solely involved coordinate transformation and value standardization, without altering the actual mesh structure. An example of face mesh before and after normalization is shown in Figure 2.

Even after removing the visibility from the data, each frame still had 1434 features, which is a quite large feature size. However, these 1434 features shared a lot of correlated information and internal connection because face muscles do not move independently but usually move as a group. In addition, each muscle is annotated to multiple landmarks, which further strengthens the correlation among the landmarks. Therefore, instead of using the normalized face mesh data to train the machine learning model, we use Principal Component Analysis (PCA) to reduce the size of the data and perform classification. The original dimensionality of the face mesh data is 478×3, which makes it very hard to train with large batch size and acceptable training time. Usually, the PCA extracts several principal components from each sample in a dataset. In our case, we applied PCA analysis to each frame of the face mesh data as the feature vector. PCA extracts the variations among various frames of face mesh, which indicates the temporal changes of the face mesh. We also trained a machine learning model that classified the face mesh data by analyzing such patterns in temporal changes (discussed later).

PCA is a widely used statistical technique that helps understand and simplify complex data sets. It provides a way to reduce the dimensionality of data while preserving as much information as possible. By extracting the most significant patterns and relationships within the data, PCA enables us to uncover the underlying structure and identify the key variables that drive the variation in the dataset. Through this process, PCA aids in visualizing and comprehending large datasets, enhancing data exploration, feature selection, and machine learning tasks. The core idea behind PCA is to find a new set of variables, called principal components, which are linear combinations of the original variables. These principal components are derived in such a way that the first component captures the maximum amount of variation present in the data, the second component captures the remaining variation orthogonal to the first component, and so on. Each subsequent component accounts for the maximum remaining variation while being uncorrelated with the previous components. In this experiment, we applied PCA to process the extracted face mesh data before training the classification model.

An LSTM network with a hidden size of 32 was applied as the classifier to perform dementia detection based on the face mesh data. Each frame of the face mesh data was first reduced by PCA into 16 principal components, which were forwarded and input into each time step of the LSTM network in time order. After the LSTM network processed the last frame of the face mesh data, the hidden states at the last time step of the LSTM network were forwarded to a dense layer. The dense layer took the hidden states of the LSTM network as its input and output a score pair as the probability of the sample belonging to either dementia or non-dementia. We used the cross-entropy loss as the loss function and the Adam optimizer with a learning rate of 0.001 to train the model.

To validate the performance of our proposed methods, we created a test set from the PROMPT dataset and ensured the following:

- The identity information of each patient was not leaked. In the data collection of the dataset, each patient paid different numbers of visits to the doctors. Therefore, each patient provided 1–10 samples in the dataset. In the creation of the test dataset, if a patient was in the test set, we put all his or her data in the test set;
- The number of samples and the number of subjects of each category were approximately equal. Because of the fact mentioned above, we also managed to keep a balance in each category of the test set.

After completing the data pre-processing and PCA reduction, we trained the LSTM network on the training data, and the performance was evaluated on the test set.

### 4.2. Experimental Settings for HOG features

We utilized the Open Face framework [40] to extract HOG features from video data in the PROMPT dataset. The Open Face approach involves several sequential steps, as outlined in [41]. First, it performs face tracking using the Constrained Local Neural Field (CLNF) method [42] as a facial landmark detector and tracker to track the face and obtain geometric features. Then, the alignment and masking step is responsible for rectifying the tracked face and compensating for variations caused by plane rotation and scaling. After the face is aligned, appearance features are extracted in the form of HOG features. This involves dividing the face into blocks of 8×8 pixels, resulting in 12×12 blocks with histograms of 31 dimensions each. Collectively, these histograms form a 4464-dimensional vector that describes the face’s appearance, which will be used as features in this section. Additionally, geometry features are extracted, resulting in 227-dimensional vectors. SVM and Support Vector Regression (SVR) are then employed to predict the occurrences and intensities of AUs based on the HOG features and geometry features. In this section, we explain the use of HOG features, and leave AUs for the next section.

We used the Open Face tool to extract HOG features from each frame of the videos in the PROMPT dataset. We adapted the model in Section 4.1 by tuning the parameters of the model to fit the HOG feature format. We used the same test described in Section 4.1. Each frame of the HOG features was input into an LSTM network in time steps, and the final hidden states after the LSTM model had processed the whole HOG feature sequence were passed to a dense layer for final classification. Since HOG features are based on local gradients, changes in lighting conditions and color can alter the gradients and subsequently impact the extracted features. Different lighting conditions can lead to variations in the appearance of edges and shapes, potentially resulting in different gradient orientations and magnitudes. This, in turn, can influence the HOG feature representations, making the HOG features likely to be biased by the lighting conditions. Due to this reason, we wanted to compare how a single frame of HOG features could detect dementia and how multiple frames of HOG features could detect dementia. This may reveal how much the HOG features were affected by the bias in the images. Therefore, we trained several networks while varying the number of frames used in the training.

### 4.3. Experimental Settings for Action Units

Open Face utilizes machine learning models to extract 17 intensities of AUs from each frame of the videos in the PROMPT dataset. The process for extracting these AU intensities using machine learning models is described in Section 4.2. To analyze the temporal behavior of these AU intensities, we followed the approach presented in [12]. We calculated the mean and variance of the 17 AU intensities over time for the entire video, which typically spanned approximately 10 min. This calculation yielded a feature vector consisting of 34 elements for each video in the PROMPT dataset. However, the PROMPT dataset consists of 447 videos from 117 subjects. Each video lasts around 10 min, with a frame rate of 30 frames per second (FPS). Consequently, we had approximately 18,000 frames per video, and each frame contained 17 AU intensities. Merely reducing the dimensionality from over 300,000 features to 34 features would discard a significant portion of the video’s information. To address this issue, we employed a segmentation method that divided each video into smaller video clips with a fixed number of frames. This segmentation allowed us to generate additional features when computing the mean or variance from the AU intensities. In this study, we explored two segmentation settings, where we divided the videos into either 1024 frames or 512 frames to capture different temporal contexts and increase the number of features available for analysis.

After extracting the mean and variance features from the PROMPT dataset, we used an SVM classifier with RBF kernel to classify the video clips into either dementia or healthy category. Since training an SVM with AU data is much faster than training an LSTM, we ran a Leave-One-Subject-Out (LOSO) scheme in the experiment. Each time, we left out all video clips of a subject as the test set, used the remaining videos as training data, and trained an SVM classifier to perform classification. After training the model, it was used to make predictions for each held-out video clip. Among all the video clips of the held-out subject, we implemented a voting system to make the final decision. We employed a threshold-based voting system in this study. Usually, majority voting is a commonly used voting strategy. It is a straightforward approach where each subject contributes multiple samples to the dataset. To determine the class of a subject, we examined the predicted class of its samples. For instance, if there were *n* samples and more than half of them were classified as dementia, we considered the subject to have dementia. Threshold-based voting is similar to majority voting, but instead of a fixed threshold of 50%, we experiment with different percentages (e.g., 25%). In threshold-based voting, if more than the specified percentage of the data (e.g., 25%) are predicted as dementia, we classify the subject as having dementia. By adjusting the threshold percentage, we can adapt the system to different scenarios. It is important to note that decreasing the threshold percentage increases recall but may lower precision. In this paper, we report the best accuracy when the threshold is optimized and the Equal Error Rate accuracy where both classes share the same error rate.

**Figure 3:**
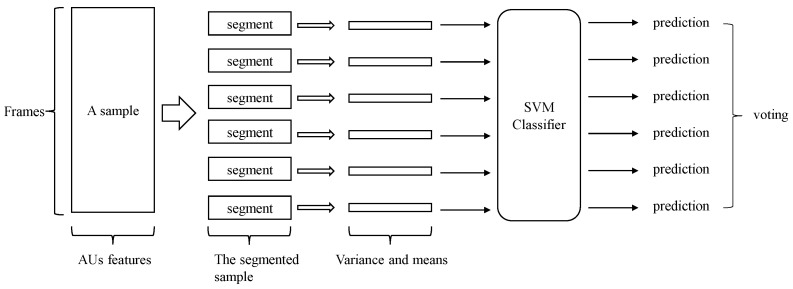
The overall pipeline of performing dementia detection using action units.

**Figure 4:**
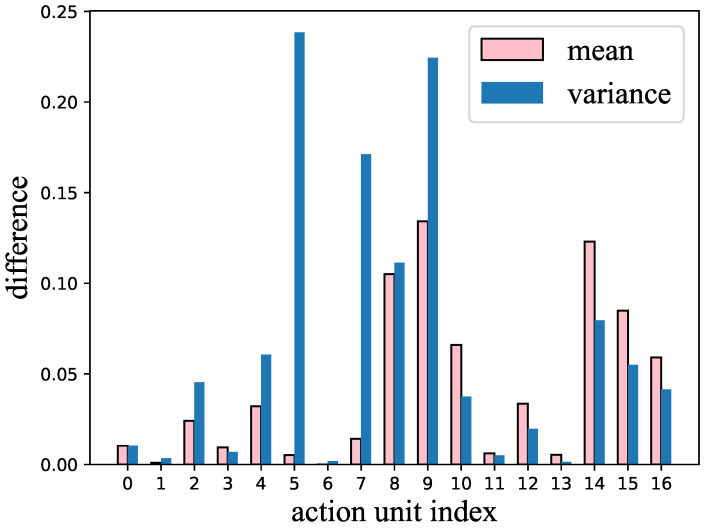
The difference in variance and mean of each action unit between healthy subjects and dementia patients.

In this experiment, we used the mean and variance of the AUs to perform the classification. The pipeline of performing dementia detection using AUs is shown in Figure 3. Figure 4 shows the difference between healthy subjects and dementia patients in terms of the mean and variance of the AU index {2,4,5,7,8,9,10,12,14,15,16}. We noticed (as shown in this figure) that not all AUs have a significant difference that includes the indicators for dementia. Therefore, we also tried using only 11 AUs that showed a significant difference in the experiment to detect dementia, discarding AUs that could mislead the classifier.

## 5. Results

### 5.1. Classification Using Face Mesh

Table 1 shows, during the PCA reduction, how much variation in the original data can be explained with different numbers of components, which also indicates how much information was maintained after the dimensionality reduction was performed. The figure shows that 99% of the information can be retained and explained by 8 principal components after performing PCA on the face mesh data. PCA reduces the dimensionality of the data that needs to be processed, requires less computation, and helps relieve the over-fitting problem. We achieved an accuracy of 66% while using the normalized and dimensionality-reduced data. As a comparison, we achieved an accuracy of 59% using the normalized face mesh data without PCA dimensionality reduction. The experiments showed that the face mesh data can potentially be employed in dementia detection. Furthermore, PCA reduction showed superior performance than its counterpart. However, even by reducing the dimension of the data, there was an over-fitting issue in the training curve, as the loss in the training data continued to decrease without improving the accuracy of the classification of the test set.

### 5.2. Classification Using HOG Features

Table 2 shows the classification performance in dementia detection when different numbers of frames of HOG features were used. From the table, we can conclude that using only 1 frame of HOG features, the model can distinguish dementia patients with an accuracy of 71.3%. Using 8 frames of HOG features improves the accuracy to 73.1%. Using 1024 frames of HOG features improves the accuracy to 79.0%. As mentioned above, HOG features are easily affected by lighting conditions. Therefore, this performance is possibly a boosted result of the bias because the healthy and dementia data were collected under different lighting conditions or different environments.

### 5.3. Classification Using Action Unit Features

Table 3 displays the classification performance of AUs in various scenarios. Our implemented voting system incorporates a variable parameter known as the threshold. This threshold represents a percentage value. During the testing phase, the tested subject has multiple video clips left out. These video clips are then classified using a trained SVM classifier. If the percentage of test video clips classified as healthy exceeds the threshold, the test data are considered to be healthy. Conversely, if the threshold is not exceeded, the subject is deemed to have dementia. In Table 3, the Max Accuracy column represents the optimal overall accuracy achieved by optimizing the threshold for best accuracy. Furthermore, the table provides the recall and precision values obtained when the threshold was optimized for the best accuracy. The Equal Error Rate (EER) Accuracy indicates the accuracy achieved by optimizing the threshold to ensure an equal error rate for dementia and healthy subjects. By segmenting the videos into 1024-frame clips, using all 17 AUs, we achieved the highest maximum accuracy of 71%. Alternatively, when we refrained from segmenting the videos and only employed 11 AUs that exhibited noticeable differences between healthy subjects and dementia patients, the best EER accuracy achieved was 68%.

### 5.4. Analysis

Table 4 summarizes the performance of different feature types using the metrics of accuracy, recall, precision, and F1 score. HOG features and AUs preferred precision over recall, while the face mesh preferred recall over precision. Even though the HOG features achieved the best accuracy performance, they were easily affected by environmental factors, which made the performance less reliable.

Table 5 shows the performance comparison between the proposed methods and other methods that are based on face images or facial features extracted from them. However, we have to emphasize that the reported performance values are based on different datasets that were collected according to different protocols. Hence, it may not be proper to compare them numerically. Even though Umeda et al. [13] reported high accuracy of 92.56% in their study, they also noted that this result was boosted by institutional bias. Their study used raw images, which are very likely to receive influence from environments such as the background or the lighting condition of the rooms where data were collected. To reduce such influence, we avoided using the raw images; instead, we extracted abstract features from the raw images and developed methods based on them. This degrades the performance but ensures better generalizability in real applications.

Table 6 shows the number of parameters and training epochs for the proposed models and some common pre-trained models. In this study, we applied PCA on the face mesh data, which significantly decreased the complexity of the LSTM network when face mesh was used, meaning we could train with larger batch sizes. When training the LSTM using the face mesh data after doing PCA, we trained with full-batch for about 338 epochs. The HOG features had a much larger input size, which made the network much more complex. When training with HOG features, we set the batch size to 64 and trained for 110 epochs.

## 6. Discussion

Our work on investigating face-related features for dementia detection significantly contributes to the advancement of artificial intelligence (AI) in healthcare, aligning with the insights provided by Jiang et al. [43] and Yu et al. [44]. In their work, Jiang et al. [43] highlighted the continuous efforts of scientists and engineers in developing AI over several decades and how AI has permeated various aspects of our lives, including healthcare. Our research specifically focuses on leveraging AI techniques to detect dementia, a critical and prevalent condition among the aging population. By exploring the potential of face-related features extracted from video data, we advance the application of AI in healthcare by introducing a non-invasive and privacy-preserving approach to early dementia screening. Our findings demonstrate that face-related features, such as face mesh, HOG features, and AUs, can serve as valuable indicators in automated computational dementia detection. Our work addresses the need for early screening methods that are less stressful, costly, and more accessible, thereby enhancing diagnosis and decision-making processes in healthcare, as highlighted by Yu et al. [44]. Additionally, our research underscores the potential of AI tools, such as the ones developed in our study, to be integrated into medicine, empowering healthcare professionals with advanced computational techniques for dementia detection and analysis.

The facial feature-based detection method proposed in our paper holds significant real-world applicability and potential usefulness in various healthcare settings. Firstly, our approach offers a non-invasive and privacy-preserving solution for early dementia screening. Unlike traditional screening methods such as blood tests or brain imaging, our method utilizes video data and facial features, which can be easily captured during routine interviews or interactions. This non-intrusive nature of our approach reduces patient reluctance and enhances accessibility to dementia detection, enabling timely interventions and treatments. Moreover, the use of facial features as indicators in automated computational dementia detection opens up possibilities for integration into existing healthcare systems. Our method can be integrated into computerized cognitive assessment tools used by healthcare professionals, assisting them in making accurate and objective assessments of dementia risk. Additionally, our approach can be deployed in telemedicine applications, facilitating remote screening and monitoring of individuals at risk of dementia, especially in underserved or remote areas where access to specialized healthcare services may be limited. Therefore, the real-world applicability of our facial feature-based detection method lies in its potential to improve dementia detection, facilitate early interventions, and enhance the overall quality of care for individuals.

Some limitations of this work include the following:

- Gathering sufficient data in the medical field poses challenges due to the sensitive nature of patient privacy. Therefore, it is difficult to amass a substantial amount of data required for training a robust and widely applicable machine learning model. Although the PROMPT dataset contains a considerable number of hours of data, it was collected from a limited group of individuals. To effectively implement this study, it is imperative to collect additional data;
- There is a need for more refined tools capable of accurately extracting facial features. While feature extraction tools such as Open Face and MediaPipe yield acceptable detection results, there are still instances of unexpected fluctuations or errors in the extracted features. Advancements in computer vision hold promise for future improvements in performance;
- The PROMPT dataset encompasses multi-modal data, including video, speech, text, and demographic information. However, this study solely focuses on the video data and does not utilize other types of data. For practical applications, it is essential to develop multi-modal approaches that comprehensively integrate various data types. This would enable a more comprehensive understanding and utilization of the PROMPT dataset.

## 7. Conclusions and Future Directions

In this paper, we investigated the potential and performance of different facial features using different methods in dementia detection. In the experiment investigating face mesh data, we found that 16 principal components can retain 99% of the information in the face mesh data. By performing the classification using face mesh data after performing PCA reduction, we achieved an accuracy of 66%. In the experiment investigating HOG features, we achieved the best accuracy of 79%. However, according to the results, there may be a potential institutional bias due to the vulnerability of HOG features to lighting conditions. In the experiment on AU data, we obtained an accuracy of 71% with proper segmentation, and we noticed that discarding some unimportant action units did not worsen the performance. Our work shows that facial features have the potential to work as an indicator in dementia detection while protecting the privacy of the patients at the same time.

For future directions, we are interested in using explainable AI to interpret the diagnosis of the classifier, which will promote the deployment of such a detecting system in real medical cases. We aim to provide examinable clues to doctors to aid them in making further and detailed analyses of the situation of their patients. Additionally, Pendrill et al. [45] revolutionized the conventional ROC curve by incorporating measurement system analysis and the Rasch model. This approach enables the unveiling of crucial insights that traditional metrics fail to capture. In the future, we intend to leverage this method in the analysis of our dementia detection results, unveiling valuable information such as task difficulty and agent ability. We believe that this information will significantly enhance and guide our research in the field of dementia detection.

## Figures and Tables

**Table 1:** Cumulative explained variance for the top 1–16 principal components.

Component Number	Cumulative Explained Variance
1 component	0.33
2 components	0.55
3 components	0.72
4 components	0.81
5 components	0.90
6 components	0.94
7 components	0.98
8–16 components	0.99

**Table 2:** Classification performance in dementia detection using different numbers of frames of HOG features.

Number of Frames	Accuracy
1 frame	71.3%
8 frames	73.1%
1024 frames	79.0%

**Table 3:** Classification performance of the action units under different settings.

Settings	Max Accuracy	Recall	Precision	EER Accuracy
All frames and 11 AUs	69%	71%	66%	68%
512 frames and 11 AUs	70%	61%	73%	67%
1024 frames and 11 AUs	70%	66%	71%	67%
1024 frames and 17 AUs	71%	50%	85%	66%
1024 frames and 17 AUs	66%	42%	77%	63%

**Table 4:** Performance of different feature types.

	Accuracy	Recall	Precision	F1 Score
Face Mesh	66%	71%	65%	68%
HOG features	79%	77%	84%	81%
AUs	71%	50%	85%	63%

**Table 5:** A performance comparison between the proposed methods and other methods that are based on face images or facial features extracted from them.

Work	Data	Reported Performance
Tanaka et al. [12]	Facial Features	82% (Area Under the Curve (AUC))
Umeda et al. [13]	Raw Face Images	93% (Accuracy) or 97% (AUC)
Ours	Face mesh	66% (Accuracy)
Ours	Action Units	71% (Accuracy)
Ours	HOG feature	79% (Accuracy)

**Table 6:** A comparison of model complexity and parameters.

Architecture	Number of Parameters
AUs with SVM	3495
face mesh with LSTM	6466
HOG features with LSTM	22.5 million
ResNet101	44.5 million
VGG16	138 million

## Data Availability

Restrictions apply to the availability of these data. Data was obtained from Keio University School of Medicine and are available at https://www.i2lab.info/resources (accessed on 13 July 2023) with the permission of Keio University School of Medicine.
